# Clinical impact and imaging results after a modified procedure of ACDF: a prospective case-controlled study based on ninety cases with two-year follow-up

**DOI:** 10.1186/s12891-021-04229-1

**Published:** 2021-07-03

**Authors:** Shunmin Wang, Jian Zhu, Kaiqiang Sun, Rongzi Chen, Jie Cao, Ruijin You, Aigang Liu, Feng Zhao, Jiangang Shi

**Affiliations:** 1 910 Hospital of China Joint Logistics Support Force, 180 Garden Road, Fengze District, Quanzhou City, Fujian Province China; 2 Department of Orthopedic Surgery, Spine Center, Changzheng Hospital, Second Military Medical University, No.415 Fengyang Road, Shanghai, 200003 People’s Republic of China

**Keywords:** Cervical spondylotic myelopathy, Modified ACDF, Axial pain, Cage subsidence, Natural height

## Abstract

**Study design:**

This is a prospective case-controlled study.

**Background:**

To analyze the postoperative axial pain and cage subsidence of patients presenting with cervical spondylotic myelopathy (CSM) after a modified procedure of ACDF (mACDF).

**Methods:**

Ninety patients with CSM were prospectively collected from 2014 to 2018. The patients were divided into spread group and non-spread group (48:42 ratio) according to the cage placement with or without releasing the Caspar cervical retractor after decompression. Spread group received conventional ACDF and non-spread group received mACDF. Patients were followed-up for at least 24 months after surgery. Radiologic data, including height of intervertebral space and Cobb Angle, were collected. Nervous system function was obtained using JOA scores, and level of pain was assessed using VAS scores.

**Results:**

A total of 90 patients were enrolled and the patients were divided into spread group (*n* = 48) and none-spread group(*n* = 42). Cage subsidence of (spread group vs none-spread group) was (0.82 ± 0.68 vs 0.58 ± 0.81) mm, (0.64 ± 0.77 vs 0.34 ± 0.46) mm, (0.48 ± 0.43 vs 0.25 ± 0.28) mm, and (0.45 ± 0.47 vs 0.17 ± 0.32) mm at 3 months, 6 months, 12 months and 24 months, respectively. The period exhibiting the most decrease of the height of intervertebral space was 3 months postoperatively. However, there was no statistical difference in the height of intervertebral space, JOA or VAS scores at the final follow-up between the two groups.

**Conclusions:**

The mACDF can avoid excessive distraction by releasing the Caspar Cervical retractor, restore the “natural height” of cervical vertebra, relieve immediate pain after surgery, and prevent rapid Cage subsidence and the loss of cervical curvature.

## Background

Cervical spondylotic myelopathy (CSM), characterized by progressive narrowing of the spinal canal and compression of the spinal cord, is a common age-related degenerative disease with the incidence of about 53.5% [1, 2],which was associated with compromised life quality and high burden of society and economy [3, 4]. Patients presenting with CSM often need surgical decompression due to its recessive onset [5]. The keys to surgical technique for CSM are decompression of the nerve roots and spinal cord, reconstruction of the stability of spinal column, and maintaining cervical alignment [6]. Anterior cervical discectomy and fusion (ACDF) has been proven to be an effective surgical method for treating patients with symptomatic CSM, since its first applied and reported by Robinson and Smith in 1958 [7]. After removal of the intervertebral disc during ACDF surgery, the interbody cage is used as a replacement, which is effective in restoring disc height. However, axial pain often occurs after surgery, and studies with long-term follow-up have revealed that complications can occur following ACDF, including the loss of cervical curvature, reduced height of intervertebral space, adjacent vertebral disease and cage subsidence [8–10]. Indirect decompression with the use of cage can be achieved by enlarging the intervertebral foramen. However, inserting too large cage can cause subsidence and bone incoherence [11, 12]. Moreover, excessive distraction by insertion of too large cage is often the leading cause of postoperative neck pain resulting from facet joint stretch or posterior neck muscle spasm [13]. And some studies have shown that the greater the cage is, the higher the risk of cage subsidence is [14]. Therefore, placing the cage of appropriate size and restoring the optimal height of intervertebral height are the main problems faced by spinal surgeons at present. Several factors may contribute to cage subsidence,including age, sex, fusion level, cage type, and bone mineral density [15]. In the past, we had to expand and enlarge the normal height of the intervertebral disc to achieve the purpose of enlarging the intervertebral foramen, but the clinical significance of this is lack of research. It is the first time, to our best knowledge, using a prospective case-control study to evaluate the postoperative complications and cage subsidence regarding the cage placement with or without releasing the Caspar cervical retractor after decompression. In the present prospective case-control study, we sought to test the discrepancies of postoperative height of fusion segment, cervical curvature, axial pain, and neurological function between the cage placement with or without releasing the Caspar cervical retractor after decompression.

## Methods

### Subjects

This study was a single center, prospective, case-control study, which enrolled patients undergoing ACDF or mACDF for treating CSM involving single-level from June 2014 to June 2018 at Naval Medical University. Patients were included in this study if they (1) were diagnosed with myelopathy or radiculopathy; (2) had compression of spinal cord or nerve root at single-level verified by Magnetic Resonance Imaging; (3) were unresponsive to conservative treatment. Patients were excluded from this study if they (1) has an increased risk of suffering from bleeding, including platelet count < 100× 109/L, and a history of surgery and substantive organ biopsy within 1 month; (2) had a life expectancy less than 90 days; (3) had spinal cord or root compression at multi-levels; (4) needed posterior approach; (5) had trauma or infection. Spread group received conventional ACDF and non-spread group received mACDF. This study was approved by the ethics committee of Second Military Medical University. There were 48 cases of intervertebral traction group (32 cases of cervical spondylotic radiculopathy. Eleven cases of mixed cervical spondylosis. Five cases of cervical spondylotic myelopathy) and 42 cases of non-traction group (29 cases of cervical spondylotic radiculopathy. Nine cases of mixed cervical spondylosis. Four cases of cervical spondylotic myelopathy).

### Selection of operative methods in ACDF

The sample size of each group was determined by the number of eligible cases during the study period, and a total of 90 patients were eventually included in the study. Prior to this, the purpose and potential risk of this study were informed, and the cage was well explained without opening the group (loosening the distractor). The size of the cage was different.

### Surgical technique

Spread group: standard general anesthesia procedure and a left-sided Smith-Robinson anterior cervical approach was used in all cases. Caspar Cervical retractor was used to distract the vertebral bodies. The anterior bony spurs were minimally excised to ensure posterior visibility and titanium cage fitting. The posterior longitudinal ligament and the posterior bony spur were removed after discectomy. The cartilage of the endplate was also removed with a curette to avoid additional damage to the endplate.

Cages were packed with the autologous cancellous bone harvested from the anterior iliac crest through a 1-cm skin incision. None-spread group: The basic surgical procedure was roughly similar to that of the spread group, except that the Caspar cervical retractor was released before placing the titanium cage, which was also known as modified ACDF (mACDF). When the Caspar cervical retractor is released and the minimum titanium cage cannot be put into the intervertebral space, expand Caspar cervical retractor and enables the minimum cage to be put into intervertebral space. After loosening the retractor and placing the interbody fusion cage, intraoperative fluoroscopy can be performed. It can also be compared with the height of the segmental intervertebral space of the lesion before operation, which can be used as a reference for grasping the tightness control depth, preventing excessive loosening and subsidence, and selecting a suitable size of cervical interbody fusion cage. If there is no fully matched interbody fusion cage, the smallest interbody fusion cage is recommended. Finally, excessive abrasion of the subchondral surface of the endplate was prevented during the operation, and the ability of postoperative interbody fusion cage collapse was reduced.

### Outcomes and imaging assessment

Patient demographics including patient age, sex, obesity, smoking, hypertension, were evaluated to figure out baseline differences between groups. Patients were followed-up for at least 24 months after surgery. Radiologic data, including 1) height of intervertebral space, 2) cervical lordosis and 3) cage subsidence were collected preoperatively, at 1, 3, 6, 12, and 24 months postoperatively.

1) The height of intervertebral space was calculated as the height of the anterior border. 2) Cervical lordosis was assessed using Cobb angle at levels C2–C7. The Cobb angle was formed by two lines, one of which is the line of the lower endplate of C2 and the other is the line of the lower endplate of C7 in a neutral position. 3) Cage subsidence was calculated as the decrease in height of intervertebral space.

**Fig. 1.**
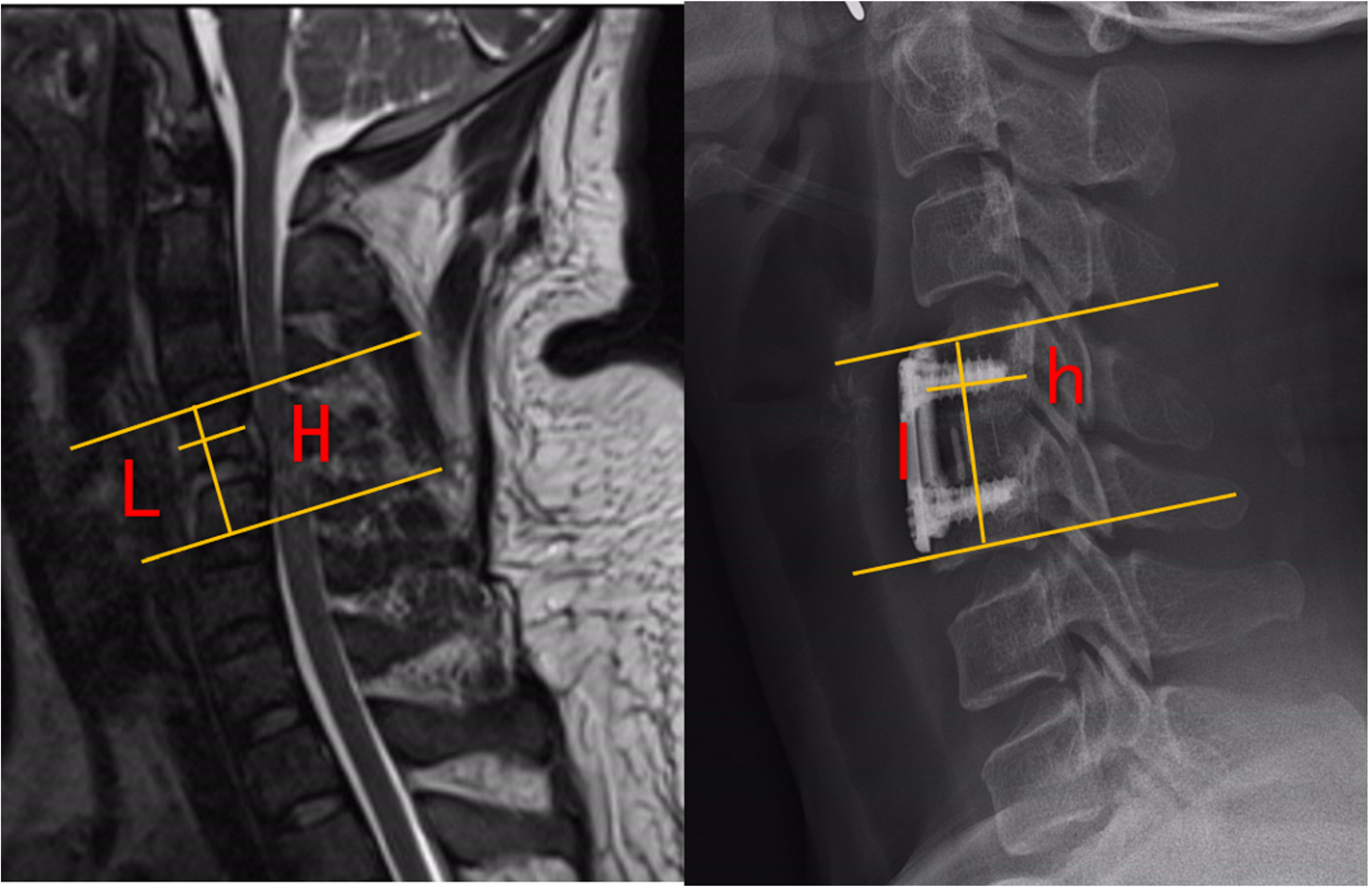
The height of intervertebral height (disk height) pre-operatively, at 1 week, and at 3, 6,12,24 months post-operatively in spread group

The absolute value of the two distances measured by MRI before operation H = the vertical distance from the upper edge of the upper vertebral body of the diseased segment to the lower edge of the lower vertebral body, L = the horizontal distance from the anterior edge to the posterior edge of the upper vertebral body of the diseased segment, Calculate R value = H/L (Fig. 1a). During the postoperative and follow-up period, only X-rays can be taken due to economic constraints. However, in order to minimize the error of the X-ray measurement, two values are measured on the X-ray film after the same method and during the follow-up: the actual measured value h and l. Calculate the value of r = L/l, which means the zoom ratio of the film. Calculate the theoretical value of H, H′ = l*R, then Δh = (h-H′)/r (if h is a positive value, it means comparing the height of the intervertebral height before and after the comparison, and vice versa, it is a decreasing value) (Fig. 1 b). This method can not only calculate the change of intervertebral height, but also calculate the settlement depth of the cage.

Nervous system function was obtained using Japanese Orthopedic Association Score System (JOA Score), and level of pain was assessed using visual analogue scale (VAS) scores preoperatively, at 1 week, 3, 6, 12, and 24 months after surgery.

### Statistical analysis

Statistical analysis was performed using the Statistical Package for the Social Sciences (SPSS) version 18.0 (IBM Armonls, NY, USA). Continuous variables were recorded as mean values ± standard deviation (SD), and categorical variables were expressed by proportions (%). The unpaired 2-tailed Student t test or Mann-Whitney U test were performed to compare the mean values or data distribution of continuous variables. And categorical variables were compared with the χ2 (chi-square) test or Fisher exact test, as appropriate. A *P* value of < 0.05 was considered statistically significant.

## Results

A total of 90 patients were enrolled in our study the patients were divided into spread group (*n* = 48) and none-spread group(*n* = 42), including 20 males and 28 females in spread group with an average age of 59.54 ± 17.65 years and 17 males and 25 females in group none-spread group with an average age of 60.07 ± 15.21 years. The demographic characteristics of patients were summarized in Table 1 and baseline characteristics were well balanced between the 2 groups, including age, gender, and basic physical condition. There were 6 cases with surgical level at C3/C4, 14 at C4/C5, 23 at C5/C6, 3 at C6/C7, and 2 at C7/T1 in spread group, while in none-spread group, 5 patients had surgical level at C3/C4, 11 at C4/C5, 24 at C5/C6, 1 at C6/C7, and 1 at C7/T1.

**Table 1:** Baseline Demographic information of patient with CSM

Variable	Spread group (*n* = 48)	None-spread group (*n* = 42)	*P* Value
Age, mean ± SD, y	59.54 ± 17.65	60.07 ± 15.21	.880
Female, No. (%)	28 (58.33)	25 (59.52)	.909
Hypertension, No. (%)	29 (60.42)	27 (64.29)	.706
Atrial fibrillation, No. (%)	18 (37.50)	17 (40.48)	.773
Smokers, No. (%)	21 (43.75)	19 (45.24)	.887
Diabetes, No. (%)	12 (25.00)	9 (21.43)	.689
Operated level, No.	48	42	
C3/C4, No. (%)	6	5	.868
C4/C5, No. (%)	14	11	
C5/C6, No. (%)	23	24	
C6/C7, No. (%)	3	1	
C7/T1, No. (%)	2	1	
Average follow-up time(m)	28.37 ± 3.60	29.82 ± 3.25	.367
Re-operation rates	8.3% (4/48)	4.8% (2/42)	0.799

**Fig. 2.**
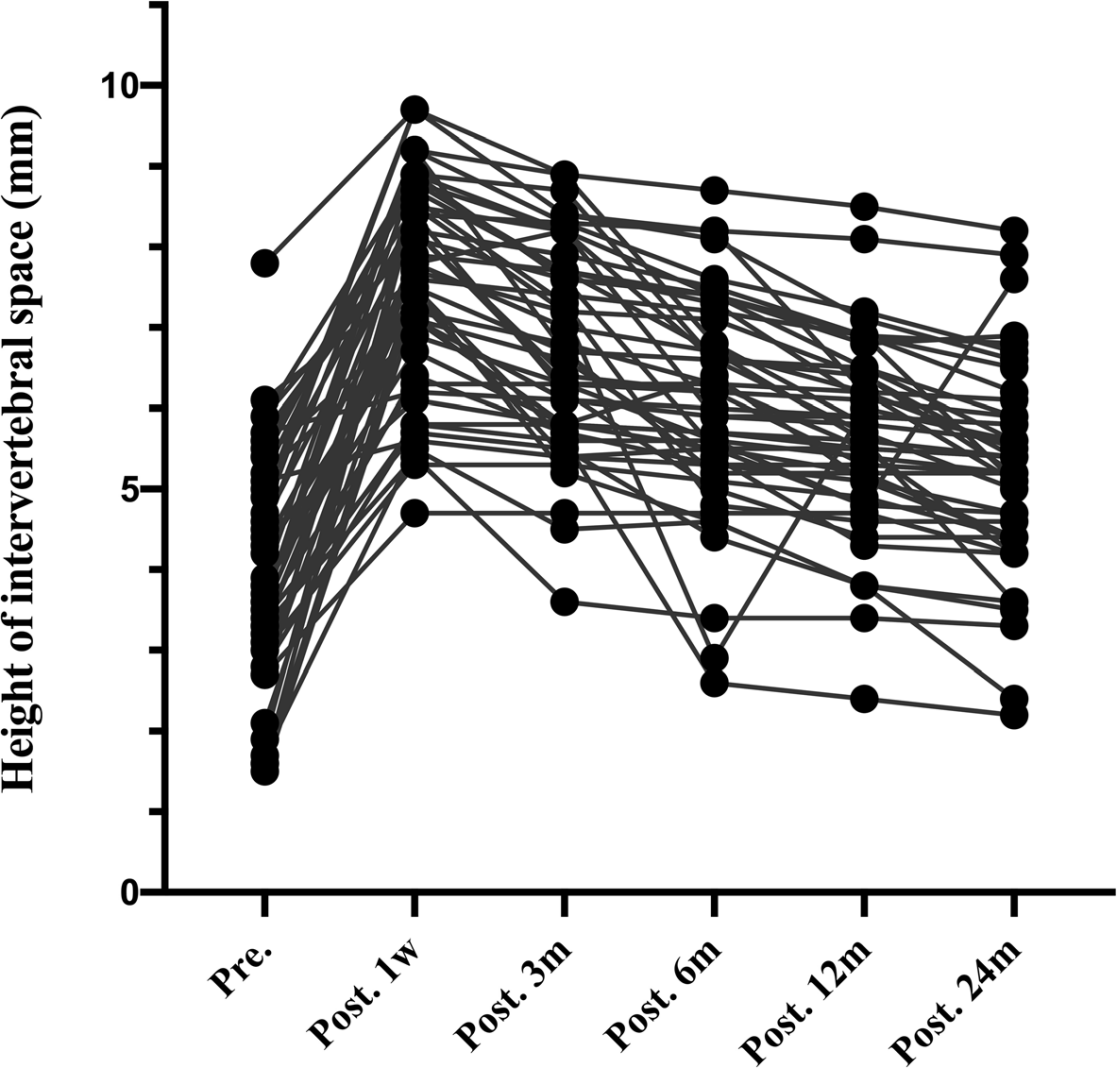
The height of intervertebral height (disk height) pre-operatively, at 1 week, and at 3, 6,12,24 months post-operatively in none-spread group

**Fig 3.**
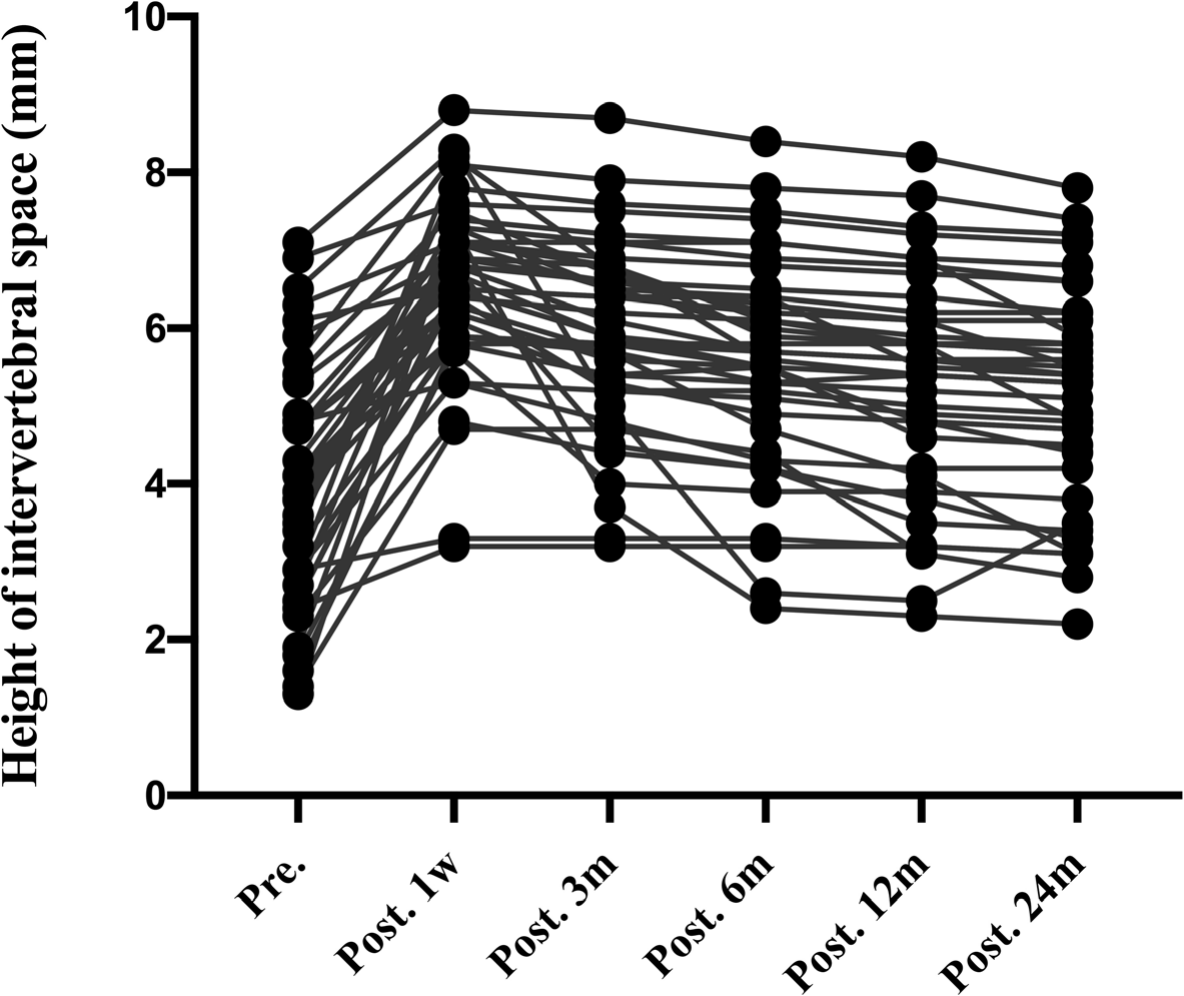
Cage subsidence at 3,6,12,24 months post-operatively between spread group and none-spread group. There was a significant difference in cage subsidence at 6),12,24 months post-operatively between the two groups (*p*<0.05)

**Fig 4.**
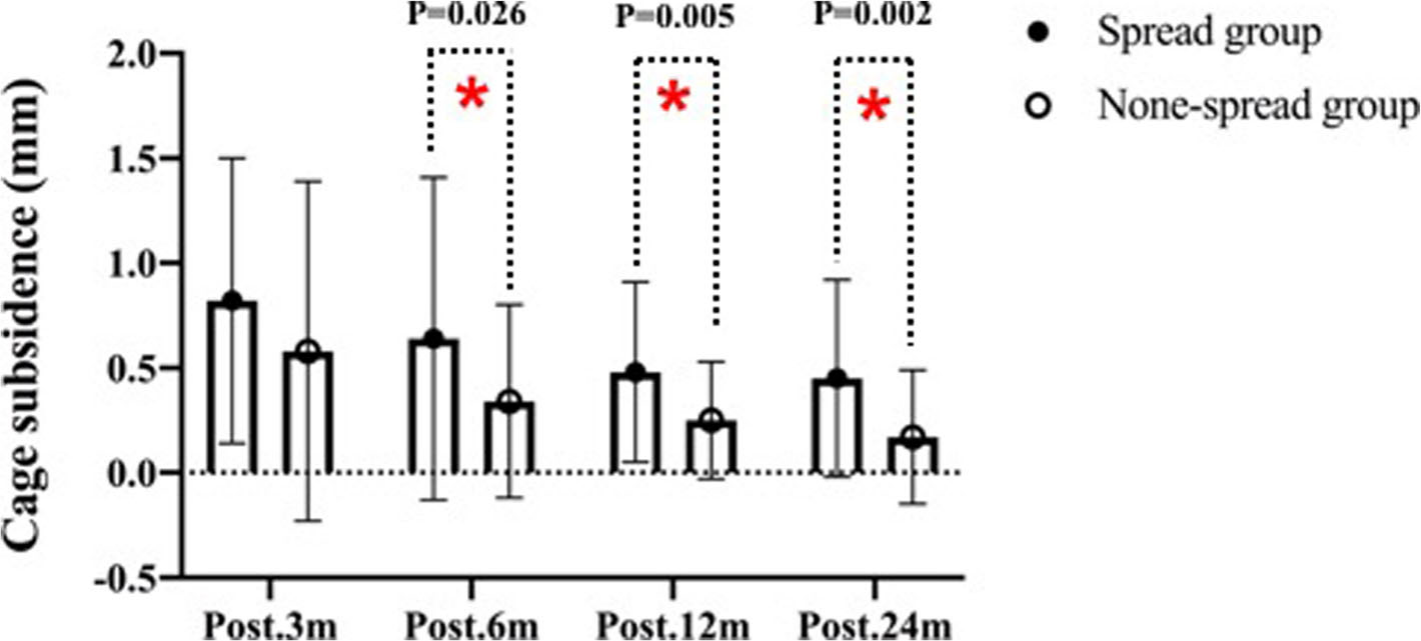
C2–C7 Cobb angle pre-operatively, at 1 week, and at 24 month post-operatively in spread group. There was a significant difference between preoperative and postoperative Cobb angle (*p*<0.01)

The imaging and clinical results are summarized in Table 2 and Table 3.The height of intervertebral space in spread group was 4.04 ± 1.41 mm before surgery, 7.59 ± 1.32 mm 1 week after surgery, 6.78 ± 1.28 mm 3 month after surgery, 6.06 ± 1.34 mm 6 months after surgery, 5.65 ± 1.18 12 months after surgery, and 5.26 ± 1.25 mm 24 months after surgery (Fig. 2). The height of intervertebral space in none-spread group was 3.94 ± 1.52 mm before surgery, 6.53 ± 1.20 mm 1 week after surgery, 5.92 ± 1.21 mm 3 months after surgery, 5.59 ± 1.34 mm 6 months after surgery, 5.34 ± 1.39 mm 12 months after surgery, and 5.16 ± 1.34 mm 24 months after surgery (Fig. 3). Cage subsidence of (spread group vs none-spread group) was (0.82 ± 0.68 vs 0.58 ± 0.81) mm, (0.64 ± 0.77 vs 0.34 ± 0.46) mm, (0.48 ± 0.43 vs 0.25 ± 0.28) mm, and (0.45 ± 0.47 vs 0.17 ± 0.32) mm at 3 months, 6 months, 12 months and 24 months, respectively (Fig. 4). The period exhibiting the most decrease of the height of intervertebral space was 3 months postoperatively. However, there was no statistical difference in the height of intervertebral space at the final follow-up between the two groups.

**Table 2:** Imaging outcomes of patients in the two groups

Variable	Spread group (*n* = 48)	None-spread group (*n* = 42)	*P* Value
Height of intervertebral space, mean ± SD, mm			
Pre.	4.04 ± 1.41	3.94 ± 1.52	.754
Post. 1w	7.59 ± 1.32	6.53 ± 1.20	< 0.01
Post. 3 m	6.78 ± 1.28	5.92 ± 1.21	.002
Post. 6 m	6.06 ± 1.34	5.59 ± 1.34	.094
Post. 12 m	5.65 ± 1.18	5.34 ± 1.39	.254
Post. 24 m	5.26 ± 1.25	5.16 ± 1.34	.724
Cage subsidence, mean ± SD, mm			
Post. 3 m	0.82 ± 0.68	0.58 ± 0.81	.127
Post. 6 m	0.64 ± 0.77	0.34 ± 0.46	.026
Post. 12 m	0.48 ± 0.43	0.25 ± 0.28	.005
Post. 24 m	0.45 ± 0.47	0.17 ± 0.32	.002
C2–C7 Cobb angle, mean ± SD, °			
Pre.	6.02 ± 9.18	6.57 ± 9.25	.778
Post. 1w	15.29 ± 8.84	10.28 ± 6.33	.004
Post. 24 m	7.29 ± 8.66	6.24 ± 6.63	.523
Average score of unoperated intervertebral disc degeneration Pfirrmann score			
Pre.	2.58 ± 0.96	2.30 ± 0.92	0.174
Post. 24 m	3.08 ± 0.84	2.38 ± 0.49	0.000

**Table 3:** Clinical outcomes of patients in the two groups

Variable	Spread group (*n* = 48)	None-spread group (*n* = 42)	*P* Value
JOA score, mean ± SD			
Pre.	11.35 ± 3.39	10.45 ± 3.20	.200
Post. 1w	14.02 ± 2.49	12.71 ± 2.87	.023
Post. 3 m	14.25 ± 2.52	13.74 ± 2.58	.344
Post. 6 m	14.88 ± 2.40	14.36 ± 2.47	.317
Post. 12 m	15.23 ± 1.89	15.10 ± 2.60	.779
Post. 24 m	14.46 ± 2.18	14.45 ± 2.74	.991
VAS, mean ± SD			
Pre.	7.21 ± 1.29	6.86 ± 1.34	.208
Post. 1w	5.96 ± 1.54	5.07 ± 1.30	.004
Post. 3 m	4.71 ± 1.37	4.74 ± 1.81	.930
Post. 6 m	4.56 ± 1.17	5.07 ± 1.96	.132
Post. 12 m	4.02 ± 0.81	3.88 ± 1.52	.581
Post. 24 m	3.46 ± 1.29	3.14 ± 1.26	.245
Complications			
PE, No. (%)	0	0	
SSI, No. (%)	2 (4.17%)	1 (2.38%)	
NI, No. (%)	1 (2.08%)	0	
SD, No. (%)	2 (4.17%)	3 (7.14%)	
Hematoma, No. (%)	3 (6.25%)	1 (2.38%)	
CSFL, No. (%)	0	1 (2.38%)	
Mortality, No. (%)	0	0	

(*PE* pulmonary embolism, *SSI* surgical site infection, *NI* neurovascular injury, *SD* swallowing discomfort, *CSFL* cerebrospinal fluid leakage)

**Fig. 5.**
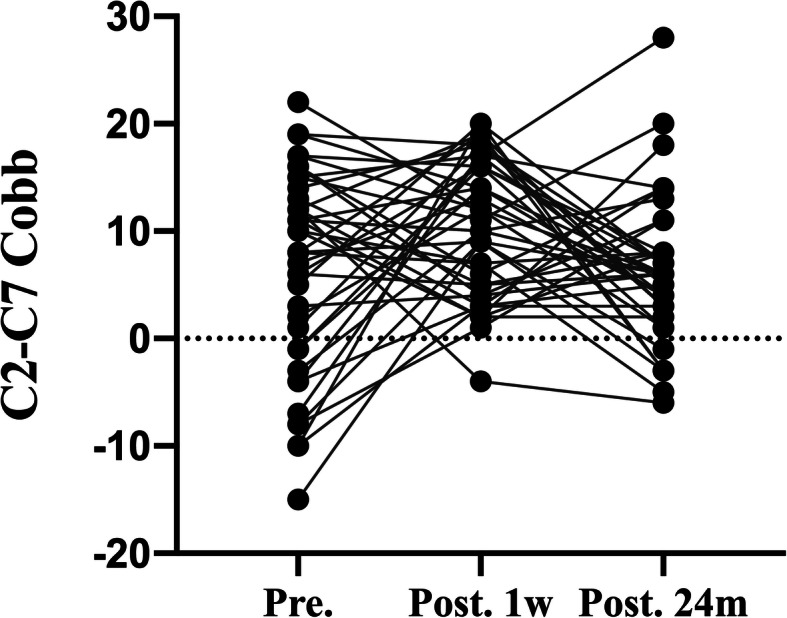
C2–C7 Cobb angle pre-operatively, at 1 week, and at 24 month post-operatively in none-spread group. There was a significant difference between preoperative and postoperative Cobb angle (*p*<0.01)

**Fig. 6.**
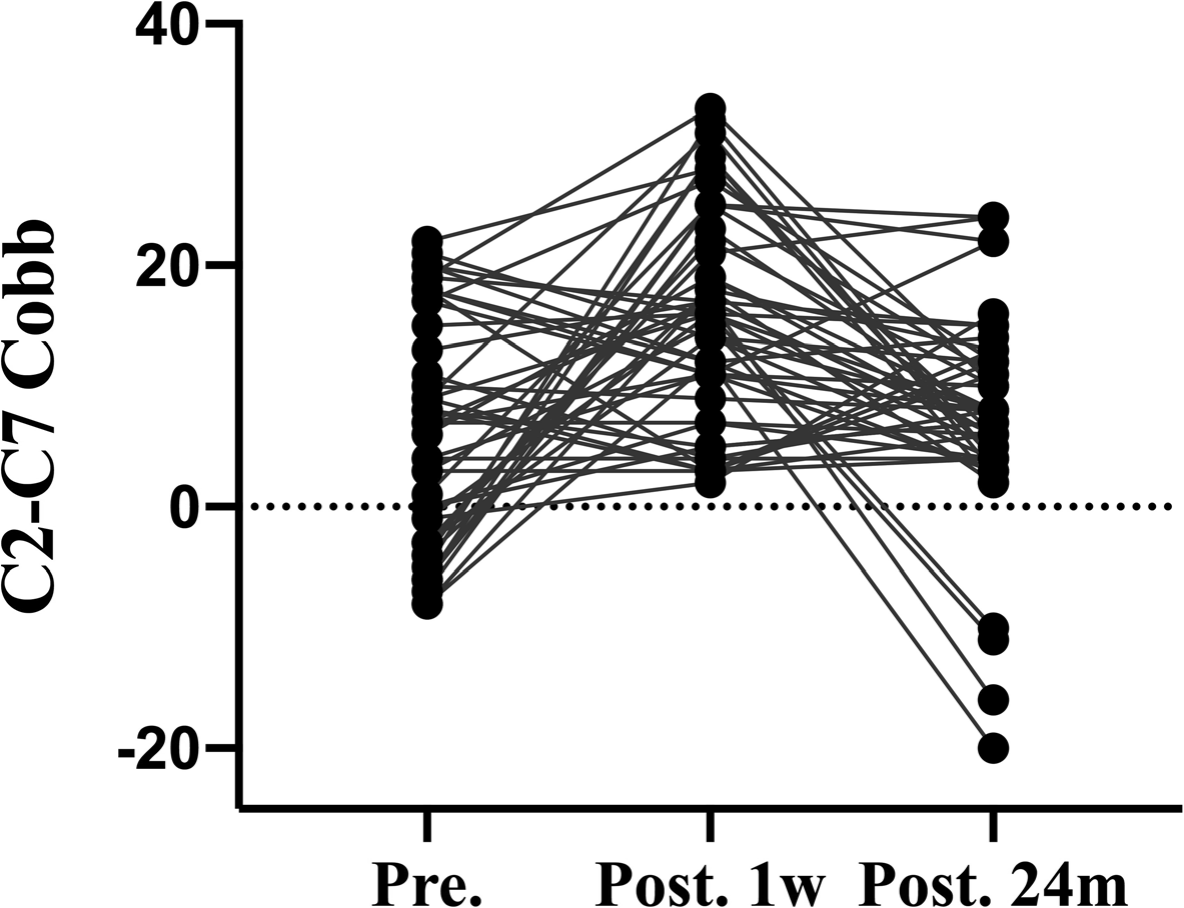
Japanese Orthopedic Association Score (JOA) pre-operatively, at 1 week, and at 3, 6,12,24 months post-operatively between spread group and none-spread group. The JOA score in spread group one week after surgery was higher than that in none-spread group (*p*<0.05). No significant difference was observed subsidence between the two groups at 3, 6,12,24 months post-operatively (*p*>0.05)

The mean ± SD of C2–C7 Cobb angle in spread group was 6.02 ± 9.18 degrees before surgery, 15.29 ± 8.84 degrees 1 week after surgery, and 7.29 ± 8.66 degrees 24 months after surgery (Fig. 5), and C2–C7 Cobb angle in none-spread group was 6.57 ± 9.25 degrees before surgery, 10.28 ± 6.33 and 6.24 ± 6.63 degrees 1 week and 24 months after surgery, respectively (Fig. 6). There was a significant difference between two groups in C2–C7 Cobb angle 1 week after surgery. The C2–C7 Cobb angle in spread group 1 week after surgery was larger than that in none-spread group with statistical significance (*p* = 0.004). However, no statistical difference was observed at the final follow-up.

**Fig. 7.**
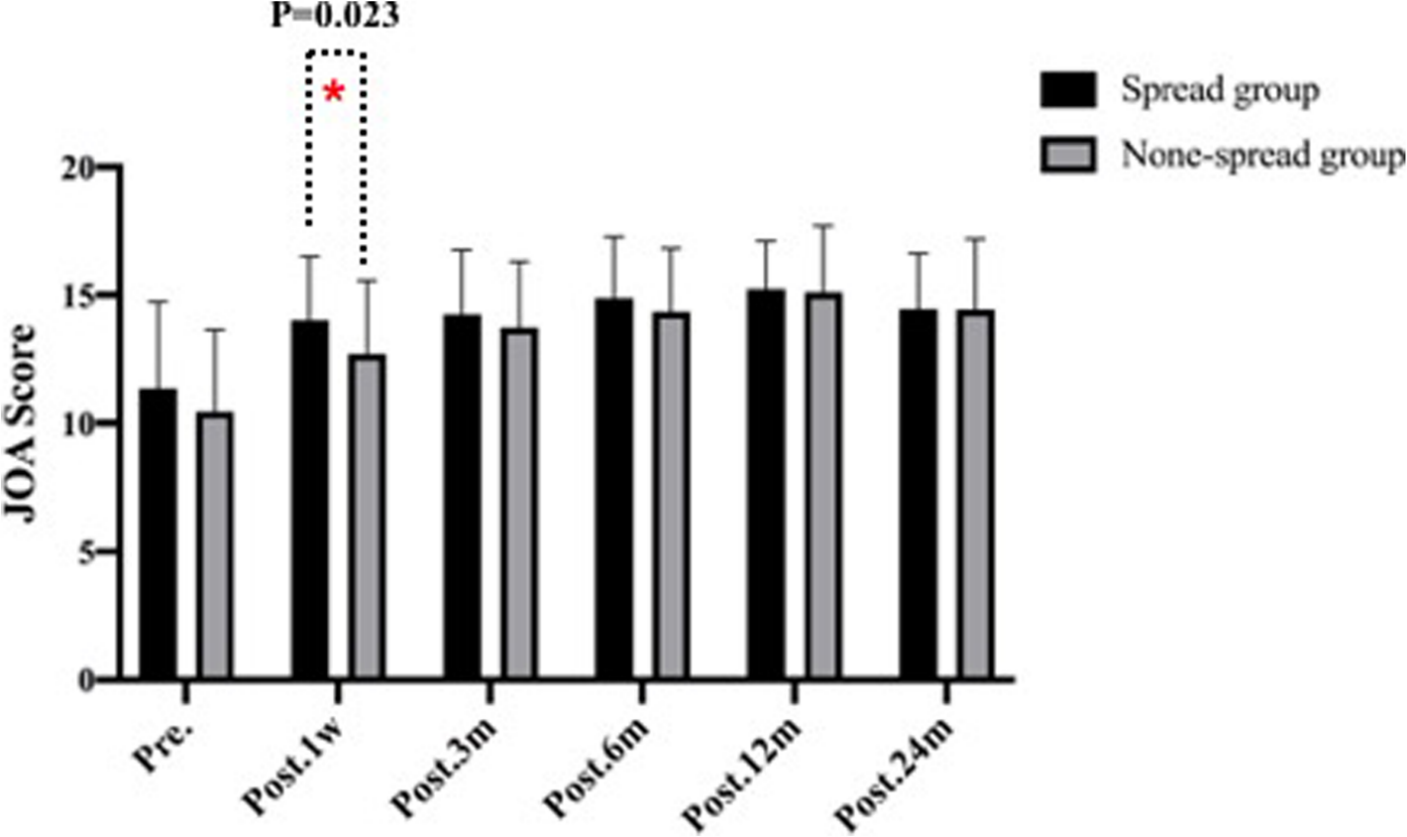
Visual analogue scale (VAS) pre-operatively, at 3, 6,12,24 months post-operatively between spread group and none-spread group. The VAS score in spread group one week after surgery was higher than that in none-spread group (*p*<0.05). No significant difference was observed subsidence between the two groups at 3, 6,12,24 months post-operatively (*p*<0.05)

**Fig. 8.**
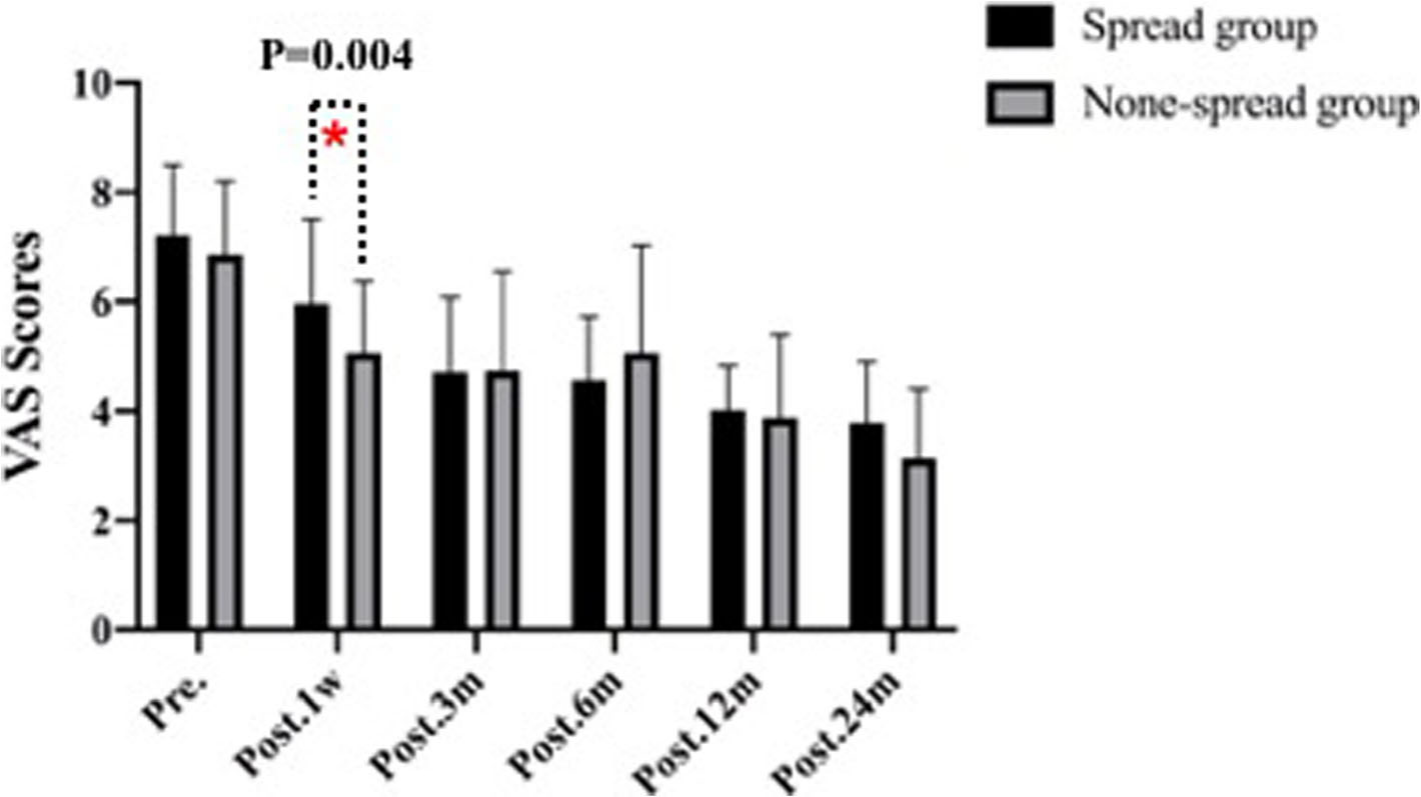
A. The absolute value of the anteroposterior diameter of the upper vertebral body of the diseased segment L and the upper edge of the upper vertebral body to the lower edge of the lower vertebral body. Absolute distance H; B. Actual measured value l and h after operation

The JOA score improved from 11.35 ± 3.39 to 14.46 ± 2.18 in spread group, and 10.45 ± 3.20 to 14.45 ± 2.74 in none-spread group (Fig. 7). No significant difference was observed in JOA score between the 2 groups. The VAS score in spread group 1 week after surgery was higher than that in none-spread group with statistical significance (*p* = 0.004) (Fig. 8). The re-operation rates were 8.3% (4/48) in spread group, and 4.8% (2/42) in none-spread group, The mean ± SD of Average score of unoperated intervertebral disc degeneration Pfirrmann score in spread group was 2.58 ± 0.96 degrees before surgery and 3.08 ± 0.84 degrees 24 months after surgery, in none-spread group was 2.30 ± 0.92 degrees before surgery, and 2.38 ± 0.49 months after surgery, respectively. (*p* < 0.001).

**Fig. 9:**
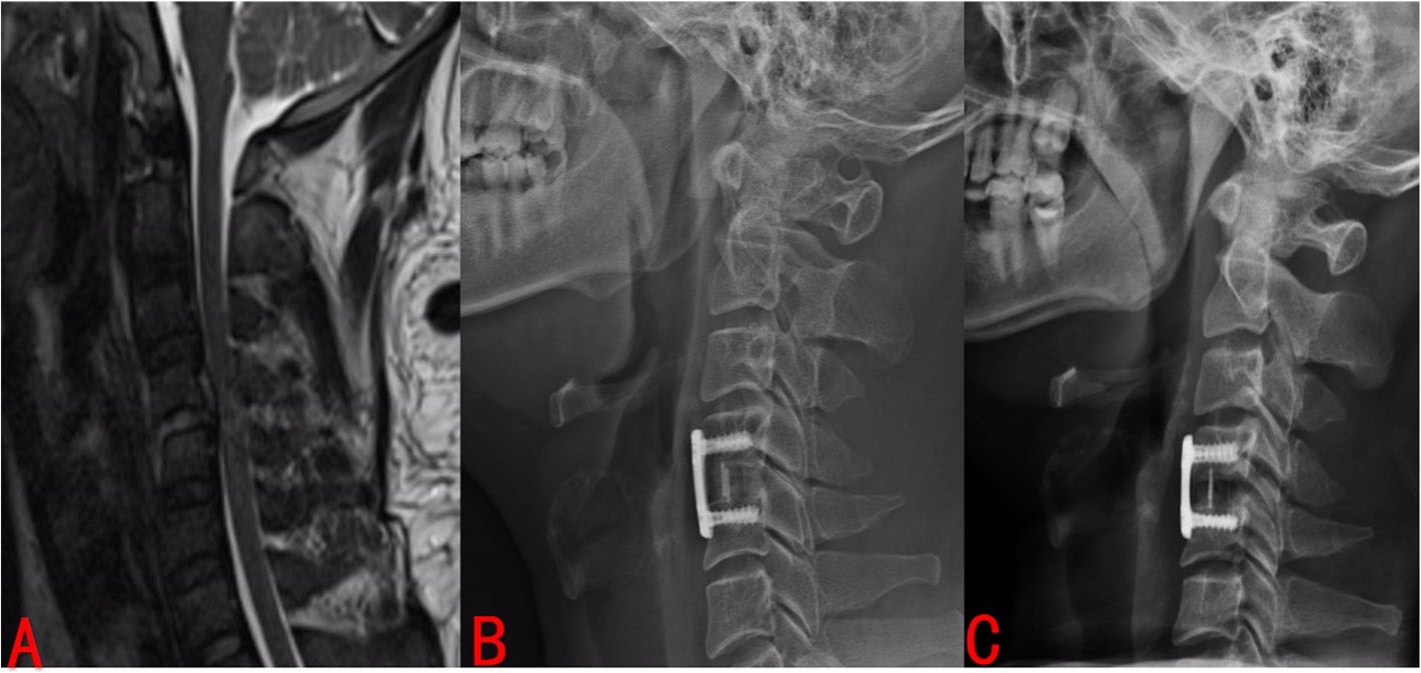
Typical case: The 60-year-old male patient was hospitalized for 2 months due to numbness in both upper limbs and unstable walking. C4/5 intervertebral disc prolapsed (Fig. 9A), and the fusion cage was implanted in the expanded state. At the last follow-up, the intervertebral disc height decreased and the cervical spine curvature became straight (Fig. 9C)

Typical case: The 60-year-old male patient was hospitalized for 2 months due to numbness in both upper limbs and unstable walking. C4/5 intervertebral disc prolapsed (Fig. 9a), and the fusion cage was implanted in the expanded state. At the last follow-up, the intervertebral disc height decreased and the cervical spine curvature became straight. (Fig. 9c).

## Discussion

The ACDF technique is a standard procedure with good clinical effect for treating patients presenting with CSM. Ylinen et al. reported that although more than half (57%) of patients recover well after surgery, many patients still suffer from chronic neck pain with reduced neck mobility [16]. Although ACDF may reduce the incidence of postoperative axial neck pain by preserving the posterior muscles, Matsumoto et al. reported that axial neck pain was observed in 25.8% of patients after ACDF during follow-up [17]. The exact mechanism of postoperative axial neck pain has not been fully elucidated. Many risk factors can contribute to postoperative axial neck, such as muscle spasm, diseases of the joints, intervertebral discs and ligaments, and vertebral instability. Facet joints are implicated as the origin of a good percentage of cervical pain [18]. Previous studies support that over-stretch of cervical facet-joint capsules may lead to cervical pain through releasing inflammatory cytokines [19, 20]. Moreover, some studies believed that excessive distraction of intervertebral space by insertion of too large cage causes postoperative neck pain due to distraction of the facet joint or spasm of the posterior neck muscles [21, 22]. However, there have been no studies regarding the cage placement with or without releasing the Caspar cervical retractor after decompression is associated with postoperative axial neck pain. In the present study, in none-spread group, due to the release of Caspar cervical retractor after decompression, the height of intervertebral space was restored to its “natural height”, cervical physiological lordosis was restored, and the height and cross-sectional area of intervertebral foramen were moderately increased [23]. An increase in intervertebral foramen cross-sectional area indicates the increased outlet space of nerve root, which may improve blood supply and reduce compression of damaged nerves. Barley et al. found that the height of intervertebral space finally stabilized, slightly higher than the preoperative height, indicating that the ideal height of cage may be associated with preoperative baseline parameters [11]. Excessive distraction of Caspar cervical retractor can result in damage to joint capsule and ligament, dislocation or subluxation of the joint, mechanical irritation or damage to the intervertebral ligaments, and ultimately axial pain [24]. If the cage is too high without releasing the Caspar cervical retractor, the pressure between the upper and lower vertebral plates will increase, which may lead to osteonecrosis, disc collapse, subsidence, and herniation, as a result of higher incidence of axial pain [25].

According to some previous studies, the incidence of subsidence is about 13.2–62.5% [26–29]. Although surgical outcomes depend primarily on adequate decompression of the spinal cord and nerve roots, postoperative radiologic changes do not directly affect the quality of life of the patients [30]. However, the loss of height of intervertebral space leads to the loss of segmental cervical curvature, affecting the cervical alignment and is thus associated with the occurrence of adjacent segmental disease. The aggravation of neck pain caused by subsidence is a major cause of poor clinical outcomes. Subsidence can result in reduced fusion rates, kyphotic deformity of the cervical spine, and reduced height of intervertebral space, eventually leading to radiculopathy [27–29]. There were similar changes in cervical curvature and height of intervertebral space in two groups. It has been reported that postoperative cervical lordosis and segmental lordosis both affect long-term clinical outcomes [31]. Although there was no statistical difference in JOA score between the two groups in our study, it may be related to the short follow-up time. Wu et al. reported that cage subsidence and cervical lordosis improvement does affect the long-term clinical outcomes [26]. In our study, changes in cage subsidence and kyphosis were higher in the spread group than in the none-spread group. Moreover, although the curvature and height of intervertebral space could be better restored after the operation without releasing the Caspar cervical retractor, JOA score in the last follow-up was not affected, but VAS score was worse. This indicates that the recovery of nerve function in the short term mainly depends on the decompression of spinal cord and nerve root, and the severity of preoperative nerve injury. The height of postoperative intervertebral space is not significantly correlated with the recovery of nerve function, which is consistent with the results reported in previous literatures [32, 33]. Besides, the greater the cage height, the greater the risk of cage subsidence. Cervical curvature is associated with degenerative changes, and studies have shown that with the improvement of cervical lordosis, height of intervertebral space increases [34]. The disc degeneration scores of the two groups are significantly different, which may be the result of a combination of natural degeneration and loss of curvature. Therefore, future studies on postoperative adjacent segment degeneration in the two groups are of more important clinical significance.

Although the cervical curvature and height of intervertebral space were better recovered immediately after the operation in spread group, it was found that the height of intervertebral space decreased too fast and finally stabilized during the follow-up, and there was no significant difference between the two groups. Subsidence is a common phenomenon and mild subsidence helps maintain internal stability. However, large subsidence must be avoided, which often results in postoperative cervical foramen stenosis and displacement. Whether subsidence affects clinical outcomes is still debatable [35, 36]. Parks et al. reported that subsidence was associated with short intervertebral foramen and poor cervical lordosis [37]. In contrast, Lee and colleagues conclude that cage subsidence does not, but segmental cervical kyphosis does affect the long-term results [38]. Truumees et al. reported that the higher the height of the cage, the higher the distractive forces and compression forces would be, leading to the greater the risk of subsidence and displacement [39]. In our cases, many subsidence occurred in the first month after surgery, and if when the placement of cage without releasing Caspar cervical retractor, axial pain occurs within a few days after the operation, and it was more likely to cause cage subsidence during long-term follow-up. In addition, the cage subsidence is often accompanied by the loss of local curvature of the cervical spine [40]. Park et al. believed that cage subsidence could lead to aggravation of local cervical curvature, but ultimately did not affect C2-C7 Cobb angle [41]. In this study, the C2-C7 Cobb angle showed a decreasing trend, indicating that the curvature of the cervical spine was straightened (two groups 7.29 ± 8.66 degrees VS 6.24 ± 6.63 degrees). However, subsidence does not necessarily affect clinical JOA scores [42]. Similarly, in our study, no significant difference was observed in JOA in the last follow-up between the two groups, but there were significant differences in VAS scores after surgery and during follow-up, which may be due to large cage and cage subsidence. In addition, many literature reports that excessive expansion may aggravate the degeneration of the intervertebral disc and affect the clinical effect [43]. Since intraoperative decompression is for better surgical vision, we cannot grasp the scale of distraction. Which may also lead to a higher rate of revisions [44]. More importantly, There was no significant difference in clinical efficacy between the two groups, and the degenerative parameters reached a balance with the preoperative baseline. Therefore, overstretching is not necessary.

This study has several limitations. The primary limitation is that this study was performed at a single center, limiting its generalizability to other centers. Another limitation of this study, like other studies, is the relatively small sample size. In addition, the patients in this study were not randomized, which may undermine the conclusions of this prospective study. However, as shown in Table 1, no difference was observed in the demographic data between the two groups. In addition, we strived to reduce systematic errors by establishing and enforcing strict inclusion and exclusion criteria to preserve sample homogeneity. To design standardized surgical procedures, the same surgical team was responsible for the same surgical instruments; Immediate postoperative symptoms were assessed by the same spine surgeon. All parameters were measured independently by three neurosurgeons who were blind to the study. Therefore, we believe that the results and conclusion of this study are true and reliable.

## Conclusions

The modified ACDF can avoid excessive distraction to some extent by releasing the Caspar Cervical retractor, restore the “natural height” of cervical vertebra, reduce the occurrence of postoperative pain in patients with CSM involving single-level, and prevent rapid Cage subsidence and the loss of cervical curvature. The necessity of restoring the normal anatomical height of the intervertebral disc is questionable.

## Data Availability

The datasets used and/or analyzed during the current study are not publicly available due to feasibility but are available from the corresponding author on reasonable request.

## Abbreviations

CSM: Cervical spondylotic myelopathy
ACDF: Anterior cervical discectomy and fusion
CT: Computer tomography
SPSS: Statistical Package for the Social Sciences
JOA: Japanese Orthopedic Association Score
VAS: Visual analogue scale
PE: Pulmonary embolism
SSI: Surgical site infection
NI: Neurovascular injury
SD: Swallowing discomfort
CSFL: Cerebrospinal fluid leakage

## References

[1] Toledano M (2013). Bartleson JD cervical spondylotic myelopathy. Neurol Clin.

[2] Shedid D, Benzel EC (2007). Cervical spondylosis anatomy: pathophysiology and biomechanics. Neurosurgery.

[3] Lau D, Chou D (2015). Mummaneni PV two-level corpectomy versus three-level discectomy for cervical spondylotic myelopathy: a comparison of perioperative, radiographic, and clinical outcomes. J Neurosurg Spine.

[4] Nakashima H, Tetreault L, Nagoshi N, Nouri A, Arnold P, Yukawa Y, Toyone T, Tanaka M, Zhou Q, Fehlings MG (2016). Comparison of outcomes of surgical treatment for ossification of the posterior longitudinal ligament versus other forms of degenerative cervical myelopathy: results from the prospective, multicenter AO spine CSM-international study of 479 patients. J Bone Joint Surg Am.

[5] Luo J, Cao K, Huang S, Yu LP, Cao T, Zhong C, Gong R, Zhou M, Zou ZY, Nong X (2015). Comparison of anterior approach versus posterior approach for the treatment of multilevel cervical spondylotic myelopathy. Eur Spine J.

[6] Matsunaga S, Komiya S, Toyama Y (2015). Risk factors for development of myelopathy in patients with cervical spondylotic cord compression. Eur Spine J.

[7] Smith GW, Robinson RA (1958). The treatment of certain cervical-spine disorders by anterior removal of the intervertebral disc and interbody fusion. J Bone Joint Surg Am.

[8] Xu J, He Y, Li Y (2020). Incidence of subsidence of seven intervertebral devices in anterior cervical discectomy and fusion: a network meta-analysis. World Neurosurgery.

[9] Ryu WHA, Platt A (2020). Deutsch H hybrid decompression and reconstruction technique for cervical spondylotic myelopathy: case series and review of the literature. J Spine Surgery.

[10] Bohlman HH, Emery SE, Goodfellow DB, Jones PK (1993). Robinson anterior cervical discectomy and arthrodesis for cervical radiculopathy. Long-term follow-up of one hundred and twenty-two patients. J Bone Joint Surg Am.

[11] Bayley JC, Yoo JU, Kruger DM, Schlegel J (1995). The role of distraction in improving the space available for the cord in cervical spondylosis. Spine.

[12] Emery SE, Bolesta MJ, Banks MA, Jones PK (1994). Robinson anterior cervical fusion comparison of the standard and modified techniques. Spine.

[13] Bai J, Zhang X, Zhang D, Ding W, Shen Y, Zhang W, Du M (2015). Impact of over distraction on occurrence of axial symptom after anterior cervical discectomy and fusion. Int J Clin Exp Med.

[14] Igarashi H, Hoshino M, Omori K, Matsuzaki H, Yamasaki K (2019). Factors influencing Interbody cage subsidence following anterior cervical discectomy and fusion. Clin Spine Surg.

[15] Karikari IO, Jain D, Owens TR, Gottfried O, Hodges RT, Nimjee MS, Bagley AC (2014). Impact of subsidence on clinical outcomes and radiographic fusion rates in anterior cervical discectomy and fusion: a systematic review. J Spinal Disord Tech.

[16] Ylinen JJ, Savolainen S, Airaksinen O, Kautiainen H, Salo P, Häkkinen A (2003). Decreased strength and mobility in patients after anterior cervical diskectomy compared with healthy subjects. Arch Phys Med Rehabil.

[17] Matsumoto M, Okada E, Ichihara D, Watanabe K, Chiba K, Toyama Y, Fujiwara H, Momoshima S, Nishiwaki Y, Hashimoto T (2012). Changes in the cross-sectional area of deep posterior extensor muscles of the cervical spine after anterior decompression and fusion: 10-year follow-up study using MRI. Eur Spine J.

[18] Cavanaugh JM, Lu Y, Chen C, Kallakuri S (2006). Pain generation in lumbar and cervical facet joints. J Bone Joint Surg Am.

[19] Cavanaugh JM, Ozaktay AC, Yamashita HT, King AI (1996). Lumbar facet pain: biomechanics, neuroanatomy and neurophysiology. J Biomech.

[20] Igarashi A, Kikuchi S, Konno S, Olmarker K (2004). Inflammatory cytokines released from the facet joint tissue in degenerative lumbar spinal disorders. Spine.

[21] Basu S, Kondety SKC (2018). Transpedicular decompression/debridement and posterior spinal fusion with instrumentation for single-level thoracic spinal tuberculosis with myelopathy-is anterior column reconstruction necessary?. Spine Deform.

[22] Ryu KS, Park CK, Jun SC, Huh HY (2010). Radiological changes of the operated and adjacent segments following cervical arthroplasty after a minimum 24-month follow-up: comparison between the Bryan and Prodisc-C devices. J Neurosurg Spine.

[23] Sun K, Sun X, Huan L, Xu X, Sun J, Duan L, Wang S, Bin Z, Bing Z, Guo Y, Shi JA (2020). Modified procedure of single-level transforaminal lumbar interbody fusion reduces immediate post-operative symptoms: a prospective case-controlled study based on two hundred and four cases. Int Orthop.

[24] Lin Z, Wang Z, Chen G, Lin T, Liu W (2020). Is facet joint distraction associated with functional outcome in patients with cervical Spondylotic radiculopathy treated with single-segment anterior cervical discectomy and fusion?. World Neurosurg.

[25] Gum JL, Reddy D, Glassman S (2016). Transforaminal lumbar Interbody fusion (TLIF). JBJS Essent Surg Tech.

[26] Wu WJ, Jiang LS, Liang Y, Dai LY (2012). Cage subsidence does not, but cervical lordosis improvement does affect the long-term results of anterior cervical fusion with stand-alone cage for degenerative cervical disc disease: a retrospective study. Eur Spine J.

[27] Gercek E, Arlet V, Delisle J, Marchesi D (2003). Subsidence of stand-alone cervical cages in anterior interbody fusion: warning. Eur Spine J.

[28] Barsa P, Suchomel P (2007). Factors affecting sagittal malalignment due to cage subsidence in standalone cage assisted anterior cervical fusion. Eur Spine J.

[29] Kast E, Derakhshani S, Bothmann M, Oberle J (2009). Subsidence after anterior cervical inter-body fusion. A randomized prospective clinical trial. Neurosurg Rev.

[30] Godlewski B, Stachura MK, Czepko RA, Banach M, Czepko R (2018). Analysis of changes in cervical spinal curvature and intervertebral disk space height following ACDF surgery in a group of 100 patients followed up for 12 months. J Clin Neurosci.

[31] Katsuura A, Hukuda S, Saruhashi Y, Mori K (2001). Kyphotic malalignment after anterior cervical fusion is one of the factors promoting the degenerative process in adjacent intervertebral levels. Eur Spine J.

[32] Yonenobu K, Abumi K, Nagata K, Taketomi E, Ueyama K (2001). Interobserver and intraobserver reliability of the japanese orthopaedic association scoring system for evaluation of cervical compression myelopathy. Spine.

[33] Kawakami M, Tamaki T, Yoshida M, Hayashi N, Ando M, Yamada H (1999). Axial symptoms and cervical alignments after cervical anterior spinal fusion for patients with cervical myelopathy. J Spinal Disord.

[34] Gao K, Zhang J, Lai J, Liu W, Lyu H, Wu Y, Lin Z, Cao Y (2019). Correlation between cervical lordosis and cervical disc herniation in young patients with neck pain. Medicine (Baltimore).

[35] Tomé-Bermejo F, Morales-Valencia JA, Moreno-Pérez J, Marfil-Pérez J, Díaz-Dominguez A, Piñera AR, Alvarez L (2017). Degenerative cervical disc disease: long-term changes in sagittal alignment and their clinical implications after cervical Interbody fusion cage subsidence: a prospective study with standalone Lordotic tantalum cages. Clin Spine Surg.

[36] Yson SC, Sembrano JN, Santos ER (2017). Comparison of allograft and polyetheretherketone (PEEK) cage subsidence rates in anterior cervical discectomy and fusion (ACDF). J Clin Neurosci.

[37] Park JH, Hyun SJ, Lee CH, Kim KJ, Yeom JS, Jahng TA, Kim HJ (2016). Efficacy of a short plate with an oblique screw trajectory for anterior cervical plating: a comparative study with a 2-year minimum follow-up. Clin Spine Surg.

[38] Lee CH, Kim KJ, Hyun SJ, Yeom JS, Jahng TA, Kim HJ (2015). Subsidence as of 12 months after single-level anterior cervical inter-body fusion. Is it related to clinical outcomes?. Acta Neurochir.

[39] Truumees E, Demetropoulos CK, Yang KH, Herkowitz HN (2002). Effects of disc height and distractive forces on graft compression in an anterior cervical discectomy model. Spine.

[40] Nakanishi Y, Naito K, Yamagata T, Masaki Y, Shimokawa N, Nishikawa M, Ohata K, Takami T (2020). Safety of anterior cervical discectomy and fusion using titanium-coated polyetheretherketone stand-alone cages: multicenter prospective study of incidence of cage subsidence. J Clin Neurosci.

[41] Park JY, Choi KY, Moon BJ, Hur H, Jang JW, Lee JK (2016). Subsidence after single-level anterior cervical fusion with a stand-alone cage. J Clin Neurosci.

[42] Kim YS, Park JY, Moon BJ, Kim SD, Lee JK (2018). Is stand alone PEEK cage the gold standard in multilevel anterior cervical discectomy and fusion (ACDF)? Results of a minimum 1-year follow up. J Clin Neurosci.

[43] Elder BD, Lo SF, Kosztowski TA, et al. A systematic review of the use of expandable cages in the cervical spine. Neurosurg Rev. 2016;39(1):1-11, 11. 10.1007/s10143-015-0649-8. 10.1007/s10143-015-0649-8 26212700

[44] Doherty RJ, Wahood W, Yolcu YU, Alvi MA, Elder BD, Bydon M. Determining the difference in clinical and radiologic outcomes between expandable and nonexpandable titanium cages in cervical fusion procedures: a systematic review and meta-analysis. World Neurosurg. 2021. 10.1016/j.wneu.2021.01.027 33516869

